# Health Tract, No. 304

**Published:** 1867-09

**Authors:** 


### HEALTH TRACT, No. 304.

### "BEVERAGES,"

So called, are the tempters to the first steps towards drunkenness. At the appropiate seasons, the newspapers abound in receipts for making various kinds of summer drinks, wines, cordials, beers, and cider. There is no easier and more certain way of making a family of drunkards than by having such things always at hand, "in case of sickness," as it is termed. I know a man, my neighbor for many years, who was accustomed to 'lay in' a barrel of cider every autumn, and it was placed on the table every day until exhausted; but every day it became more sour, alcoholic; and by the time it was out, the stimulus of it was so decided that a disagreeable want was experienced, and it was determined that next year he would lay in two barrels; at length six barrels were laid in for the winter's supply; meanwhile, my friend and neighbor had become a habitual drinker, on rising, at breakfast, at dinner, in the middle of the afternoon and from supper until late bed-time; cider is too tame now; his position and means demand 'and supply the costliest brandies; he is seldom drunk, but always full; there does not live anywhere a more honorable and high-minded man; in all business transactions he has maintained the very highest position for incorruptible integrity, and as a neighbor and friend and good citizen, he has no superior; but take from him the brandy bottle for a day, and he would go mad, or die of exhaustion—of an insufferable sinking.

It is an incontrovertible physiological fact, that any artificial stimulus continued for a few days, makes the system feel the want of it, instinctively lean upon it, and look for it; but this is not all; the same amount of stimulation is demanded every day; but to create that amount, a larger and an increasing quantity of the stimulus becomes necessary, or it must be more frequently supplied. No habitual user of spirits, of tea and coffee, can possibly deny this, after ten year's practice; as proof, see how much oftener they drink or smoke or chew than when they first entered on the miserable, useless and degrading career of self-indulgence. The truth is, there is no safety except in absolute refusal even to taste a drop or chew an atom. He who takes one drop may die in the gutter; he who has the high moral courage to refuse that first drop, that first atom, never can!

I know a whole family of beautiful grown-up daughters, not one of whom by any chance ever refuses, at home or at a party or on a picnic, to take a glass of brandy, toddy or any of its likes. The habit was formed by the mother making brandy the panacea for every stomach-ache, for nasuea, for faintness, for bodily derangement. for a chill, for an over-work or an over-meal.—FROM THE BOSTON WATCHMAN & REFLECTOR.

